# A Case Report of a Rare Pediatric Brain Tumor: Congenital Glioblastoma

**DOI:** 10.7759/cureus.23229

**Published:** 2022-03-16

**Authors:** Antonio G Junior, Nina M. P. Abreu, Marcus Vinícius B. Leal, Hilanne L. A. de Aquino, João Paulo C. Rodrigues, Caio B. Malveira, Yael P. Silva, Pablo P. A. Coimbra

**Affiliations:** 1 Radiology, Icahn School of Medicine at Mount Sinai, New York, USA; 2 Department of Radiology, Antonio Prudente Hospital, Fortaleza, BRA; 3 Private Practice Physician, Buriti Hospital, Goiânia, BRA

**Keywords:** childhood tumors, parenchymal hemorrhage, congenital glioma, brain tumor, glioblastoma

## Abstract

Congenital brain tumors are extremely rare; even with advances in prenatal imaging tests, it is still uncommon. Congenital glioblastoma (GBM) is a rare childhood tumor. With less than 50 cases described in the literature, it corresponds to less than 2% of tumors in children under two months of life. Moreover, it has a markedly poor prognosis due to the risk of intracranial hemorrhage, especially during surgical resection. This study reports the case of a 20-day-old asymptomatic child who presented with increased head circumference during a pediatric routine check-up. A transfontanellar ultrasound was performed, exhibiting hydrocephalus, large parenchymal hemorrhage, and expansive formation. Magnetic resonance imaging pointed to a massive infiltrative lesion, with heterogeneous enhancement, delimiting central areas of necrosis with hematic material inside, associated with a compressive effect on the adjacent parenchyma. Additional histopathological analysis, immunohistochemistry, and DNA methylation test confirmed the diagnosis of GBM. The patient was submitted to surgical intervention and chemotherapy, achieving a 26-month-old survival by the time this study was written.

## Introduction

Congenital brain tumors are very rare; even with advances in prenatal imaging tests, it is still uncommon [1]. Central nervous system (CNS) tumors incidence ranges from 1.1 to 3.6 episodes in 100,000 newborns and is responsible for 0.04-0.18% of deaths among the pediatric population in the first year of life [2-5].

Glioblastoma (GBM) multiform (grade IV according to the World Health Organization (WHO) classification of brain tumor) is the most frequent malignant brain tumor within the adult population [6]. On the other side, GBMs are infrequently diagnosed as congenital CNS tumors [1,3,7]. It comprehends about 2-9% of all congenital CNS tumor cases [8]. The congenital and adult forms of GBMs share histological characteristics, such as necrosis and endothelial proliferation [6,9].

Usually, it is diagnosed during the perinatal period via routine fetal ultrasound around 28-39 weeks [3]. Although poorly described in literature due to its rarity, congenital CNS tumors are mostly described as being supratentorial, contrasting the preferred infratentorial site of other pediatric brain tumors [9,10].

According to a systematic review performed by Hou et al., 40% of 35 patients were stillborn or expired within one week of life, and the mortality rate at two months of age increased to 60%. Patients who were submitted to surgical intervention with or without adjunctive therapy had a mean survival time equal to or above 96.5 weeks. The long-term prognosis is poor due to the risks of intracranial hemorrhage, especially during surgical resection [3]. Our article reports the case of a child diagnosed with congenital GBM who achieved a 26-month survival.

This case report was previously exhibited as an oral presentation at the 2021 American Society of Neuroradiology Annual Meeting Congenital on May 26, 2021.

## Case presentation

**Figure 1:**
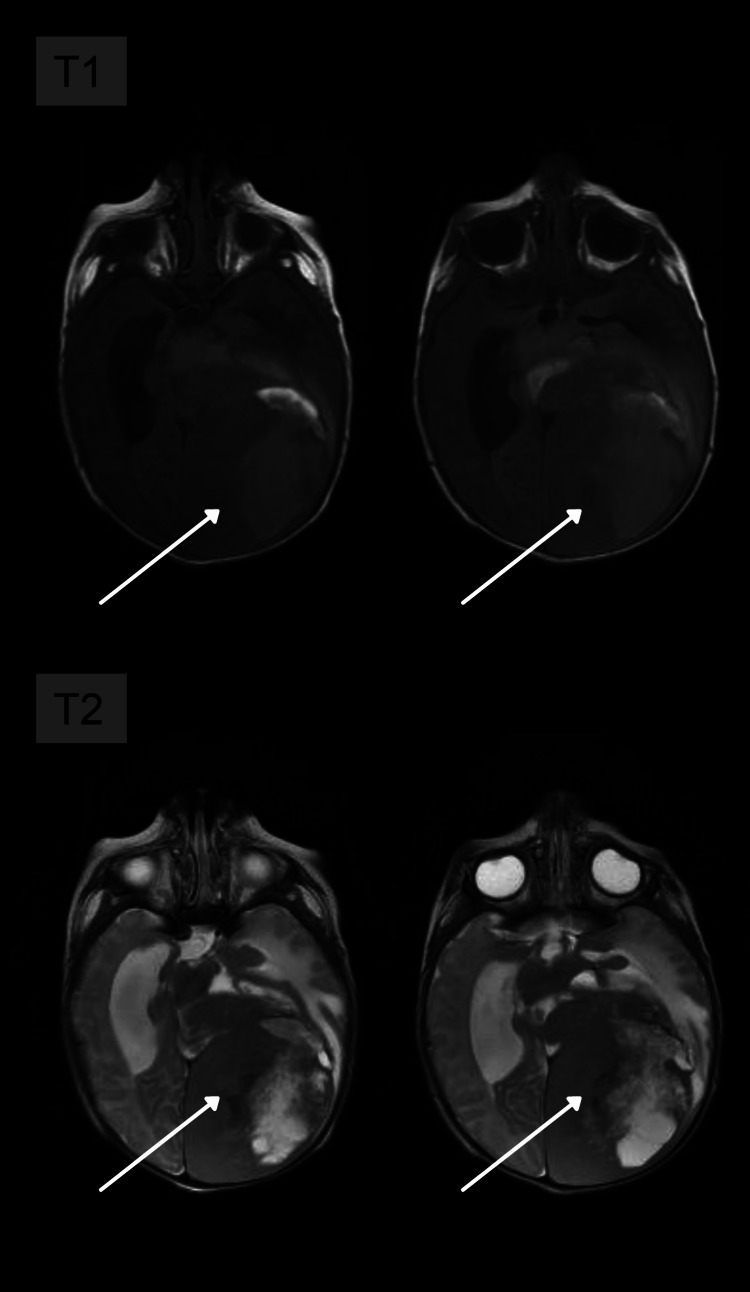
T1-weighted axial images showing a large expansive hemispheric lesion on the left, temporoparietal, heterogeneous, imprecise contours, with areas of high signal, probably correlated with hemorrhagic content. T2-weighted axial images, with a very heterogeneous lesion matrix, as well as areas of the hyperintense signal that may be associated with necrosis or cystic degeneration.

**Figure 2:**
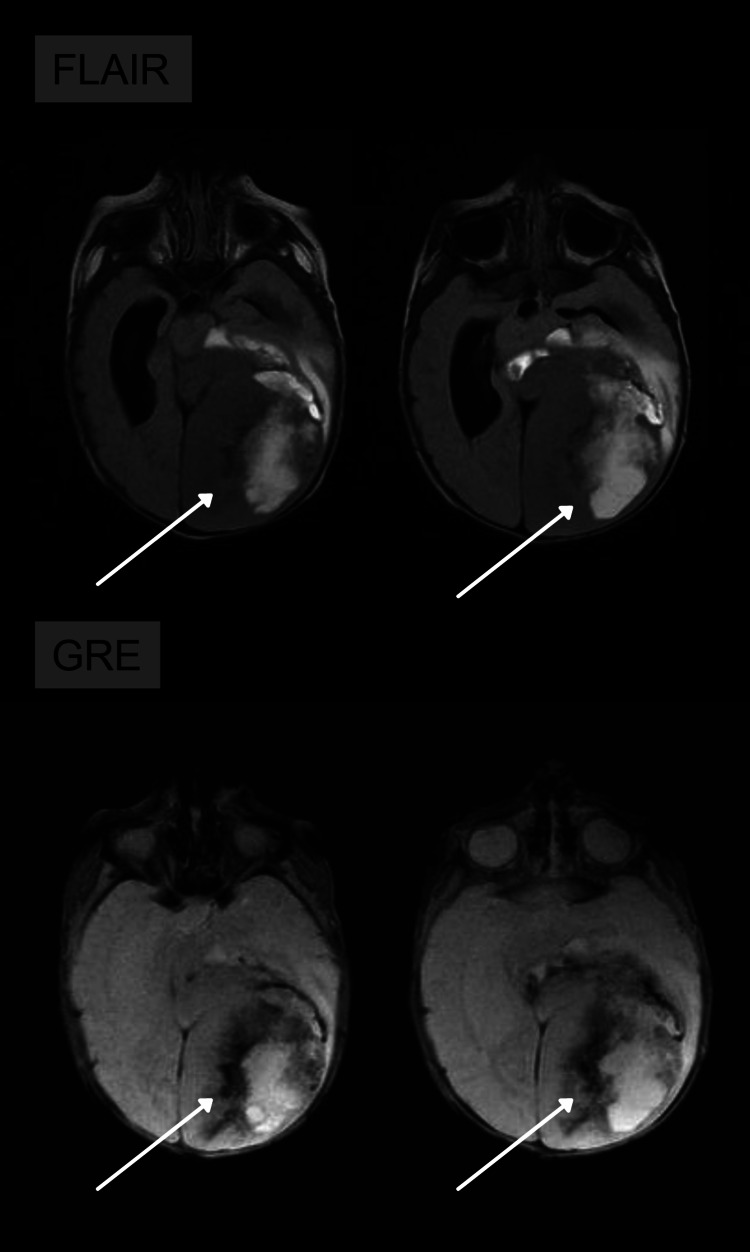
FLAIR: axial images in the FLAIR sequence show an increase in diffuse signal both in the tumor area and in the edema of the white substance, indicating a high degree of hydration of the lesion. GRE: axial images in the GRE sequence showing areas of hypointense signal, which may correspond to calcification or hematic component. GRE: gradient-echo sequences; FLAIR: fluid-attenuated inversion recovery

**Figure 3:**
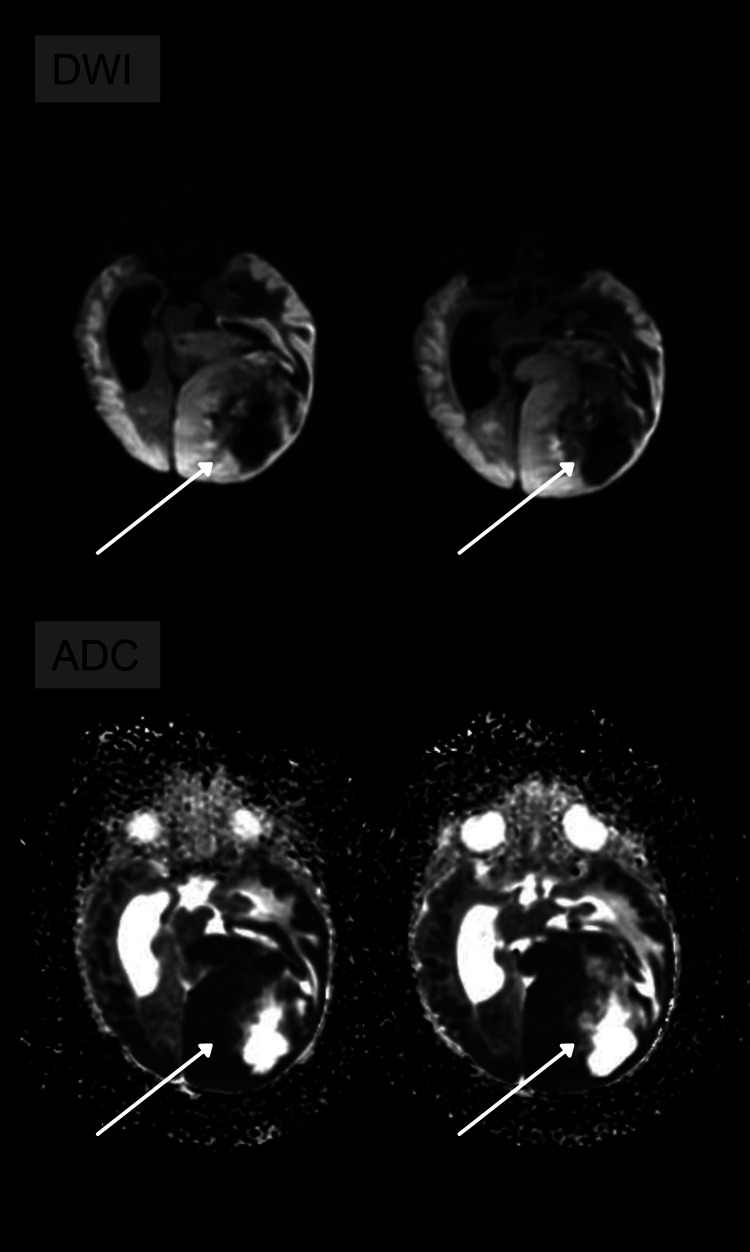
Heterogeneous areas of diffusion restriction are noted on the DWI and ADC map, indicating the high cellularity of the lesion. ADC: apparent diffusion coefficient; DWI: diffusion-weighted imaging

A 20-day-old child, born full-term without any complications, was asymptomatic when an abnormal increase in head circumference was verified during a pediatric routine check-up. A transfontanellar ultrasound was performed and hydrocephalus, extensive parenchymal hematoma, and expansive formation were observed. Magnetic resonance imaging (MRI) was performed to optimize the evaluation of the findings. MRI revealed intra-axial, temporoparietal expansive formation, with areas of low signal in the gradient sequence (which may correspond to hematic component and/or calcification), as well as areas of high signal on T2 (compatible with cystic degeneration and/or necrosis), circumjacent vasogenic edema, with heterogeneous and predominantly peripheral enhancement after contrast administration, affecting the left occipital lobe, measuring approximately 6.8 cm craniocaudal (CC) × 6.0 cm anteroposterior (AP) × 5.8 cm laterolateral (LL). It exerted a marked mass effect, characterized by compression of the corresponding lateral ventricle posterior horn as well as the ipsilateral midbrain. In addition, there was moderate ectasia of the supratentorial ventricular system (Figures 1-3). Immunohistochemistry and methylation of tumor DNA were performed, which were compatible with GBM.

The patient was submitted to surgery, followed by histopathological analysis, which suggested a dysembryoplastic neuroepithelial tumor, but requested additional immunohistochemistry to aid in the diagnosis. The panel result was positive for glial fibrillary acidic protein (polyclonal), integrase interactor 1 (MRQ-27), S-100 (polyclonal), CD34 (clone QBEND-10 in vascular endothelial cells), and Ki67 (clone 30-9) score was 70%. Synaptophysin was negative (clone SY38). Additionally, DNA methylation was executed, and, together, the results of these tests were compatible with GBM.

After surgery, the patient was placed on chemotherapy. The expansive lesion enlarged since the first MRI, reaching 12.0 cm × 9.6 cm × 11.0 cm (CC × AP × LL) by 25 days post-operation, and regressed to 9.0 × 8.9 × 5.8 cm (CC × AP × LL) eight months posterior to surgical intervention and chemotherapy.

## Discussion

Congenital GBM can be classified according to the time of diagnosis into three groups: definitely congenital, probably congenital, and possibly congenital, proposed by Solitare and Krigman [7,11,12]. Our patient can be categorized into the category “probably congenital” because the first abnormalities compatible with GBM were only noticed within the third week of life. The tumor location was supratentorial, which is compatible with what has been outlined over the years since it was first described by Holt in 1917 [13].

Patients diagnosed with GBM after birth frequently detain symptoms of increased intracerebral pressure. In our patient’s case, the clinical symptoms that predominantly raise suspicion of altered intracranial pressure include the abnormal head circumference, followed by lethargy and vomiting [12]. Regarding imaging findings, the large, heterogeneous, bulky formations with a predilection for supratentorial location are usually observed, associated with hematoma and adjacent edema on congenital GBM [8,10,14]. The patient’s symptoms and imaging alterations were fundamental to guide the medical conduct, which requested additional tests such as immunohistochemistry. The staining pattern reported in the literature demonstrates strong positivity of GFAP and vimentin [5].

Before performing the MRI, the patient was submitted to a transfontanellar ultrasound. The anomalies observed were not discordant to what has been published in case reports. Identification of a heterogenous mass sometimes associated with hemorrhage and/or hydrocephalus are the primary sonographic findings of GBM [3,5,10].

MRI showed voluminous infiltrative lesion, with heterogeneous enhancement, delimiting central areas of necrosis, as well as the presence of an internal hematic substance, involving a large area of the left cerebral hemisphere, associated with substantial mass effect of the adjacent parenchyma (Figures 1-3). These characteristics are particularly crucial to distinguish GBM from another pediatric tumor with similar imaging features, Desmoplastic infantile tumor (DIT) because both are generally supratentorial and have cystic and solid components. However, restricted diffusion and heterogenous enhancement are fundamental in the recognition of these two tumors. Bader et al. evaluated 70 cases of pediatric brain tumors and found that contrast enhancement was heterogeneous in 30% of GBM cases and homogenous in 84% of DIT cases [15].

Our patient continues chemotherapy, with steady lesion progression and stable clinical condition, with a 26-month survival. Routinely, the overall prognosis is quite poor, with some studies stating an average survival of two months, even with chemotherapy and maximum surgical resection being performed [8,14,16]. Winters et al. reported positive outcomes of chemotherapy, whose patient survived over five years after surgery. On the other hand, Hou et al. reported a 21-week survival after surgery followed by chemotherapy. It is unclear if chemotherapy provides any additional benefit after surgical intervention on survival rates [3,5].

## Conclusions

Improvements in perinatal care over the past few years have enabled premature diagnosis. Nevertheless, this advance was not reflected in clinical management, mainly because of this condition’s rarity, which limits studies and consequently the development of treatment protocols focused on improving patients’ survival rates and quality of life. A lot remains to be done to be sure of what can truly benefit patients. Therefore, bringing another case to light may help foster future research initiatives.
